# Evaluation of Different Terrestrial Oils as an Alternative to Dietary Fish Oil on Feed Physical Properties, Growth, Feed Utilization, and Fatty Acid Profile of Gangetic Catfish (*Mystus cavasius*)

**DOI:** 10.3390/ani16020330

**Published:** 2026-01-21

**Authors:** Sadia Taslim Helen, Tanwi Dey, Anwesha Bharoteshwari, Kazi Rakib Uddin, Muhammad Anamul Kabir, Md. Rakibul Hasan, Md. Sakhawat Hossain

**Affiliations:** 1 Department of Aquaculture, Faculty of Fisheries, Sylhet Agricultural University, Sylhet 3100, Bangladesh; 2 Institute of Technology Transfer and Innovation, Bangladesh Council of Scientific and Industrial Research (BCSIR), Dhaka 1205, Bangladesh

**Keywords:** Gangetic catfish (*Mystus cavasius*), fish oil replacement, terrestrial oils, black soldier fly larvae oil, growth performance, fatty acid composition

## Abstract

The global demand for fish oil (FO) is increasing while its supply is decreasing, which has limited its use in aquafeeds. Research on alternative terrestrial oils (TOs) for commonly cultured fish species in Bangladesh is limited. This research involved a 70-day feeding experiment to assess the effectiveness of replacing FO with TOs in the diet of Gangetic catfish (*Mystus cavasius*). Five diets were formulated: a control diet (D1) with fish meal and FO, and four diets replacing FO with soybean oil (D2), black soldier fly larvae oil (D3), palm oil (D4), or a mixed oil combination (D5) of 50% black soldier fly larvae oil, 25% soybean oil, and 25% palm oil. A total of 675 fish (0.5 g each) were distributed in 15 100 L aquariums (45 fish/aquarium) and fed to satiation twice daily. Fish fed with Diet D5 showed significantly higher growth, followed by those fed with D3, D4, and D1, while D2 resulted in significantly lower growth. Fish on the D5 diet consumed the most feed, followed by those on the D3 and D2 diets, with similar feed intake levels for those on the D1 and D4 diets. FCR, FCE, and PER were not significantly affected by dietary oil sources. Whole-body lipid content (*p* < 0.05) was significantly lower in the D3 group and higher in the D2 group, while other groups showed intermediate values. The fatty acid composition in the fish reflected their diets: significantly higher n-3 LC-PUFA (EPA + DHA) content was observed in the D1 group, followed by the D4 and D3 groups, and fish fed with D2 and D5 showed significantly lower values. Alpha-linolenic acid C18:3n-3) was significantly higher in the D2 group, followed by the D3, D1, and D4 groups, with the D5 group having a significantly lower value. Total MUFA was significantly higher in D4, followed by D1, D5, and D3; the D2-fed group showed a significantly lower value. Lauric acid (C12:0) was significantly higher in D3, followed by D5; other groups showed significantly lower values. Feed physical properties were significantly influenced by oil type, with water stability, pellet durability, and palatability being significantly highest in the D2 and D5 diets, followed by D3 and D4, with D1 being the lowest. Fish on the D1 and D5 diets had a significantly higher condition factor (CF) compared to fish on the D2 diet. Considering the growth and overall performance in the current study, we concluded that under the current dietary composition, TOs can effectively replace FO in the diets of Gangetic catfish, with mixed oils, black soldier fly larvae oil, and palm oil being the most promising alternatives.

## Simple Summary

This study tested whether terrestrial oils (TOs) can replace fish oil (FO) in the diet of Gangetic catfish (*Mystus cavasius*). Over a 70-day feeding trial, fish were given five different diets: one with FO and four with TOs (soybean oil, black soldier fly larvae oil, palm oil, and a mix of black soldier fly larvae, soybean, and palm oils). The mixed oil diet led to the best growth, followed by the diets with black soldier fly larvae and palm oils. Soybean oil alone resulted in the poorest growth. While growth and body fat content varied, feed efficiency was not affected. Fish fed with FO had the highest levels of heart-healthy fats, while those fed with black soldier fly larvae and palm oils also showed good results. Linolenic acid was the lowest in the mixed oil diet, and lauric acid was the highest in the black soldier fly larvae oil group. Feed quality was best in the soybean and mixed oil diets. This study concludes that TOs, especially mixed oils, black soldier fly larvae oil, and palm oil, can successfully replace FO in the diets of Gangetic catfish.

## 1. Introduction

Aquaculture has become an essential part of the global food supply, significantly contributing to meeting the rising demand for fish. It serves as an important source of protein and vital nutrients worldwide [1]. The Food and Agriculture Organization (FAO) reports that aquaculture now supplies nearly half the fish consumed worldwide, underlining its critical role in addressing seafood demand. The population across the globe is expected to increase from 7.8 billion to 9 billion by 2050 [2]; food production must increase by 25% to 70% to meet the needs of an additional 1.2 billion people [3]. Aquaculture continues to be the fastest-growing sector in food production [4]. Meeting future demands will be challenging due to declining natural resources and competition for agricultural inputs. Subsequently, the surge in demand will require not only more products but also a higher standard of quality [5]. To meet these challenges, the aquaculture industry must develop feed formulations that are nutritionally balanced to promote the growth, health, and well-being of farmed species.

A key factor in aquafeed formulation is providing a well-balanced nutrient profile and appropriate energy content for the target species. Dietary lipids are also essential for various benefits, including being a building block of cellular membranes and a facilitator of the absorption of lipophilic nutrients [6]. Historically, dietary fish oil (FO) has played a crucial role in aquafeeds, facilitating optimal growth, enhancing feed efficiency, and influencing the fatty acid profile in farmed fish [7]. FO is considered the optimal lipid source in aquafeeds due to its balanced fatty acid composition, including long-chain poly-unsaturated fatty acids (LC-PUFAs) such as EPA and DHA, which are essential for the growth and health of fish. Additionally, these LC-PUFAs have numerous positive health impacts on humans, boosting the nutritional benefits and consumer appeal of edible fish tissue. However, the global supply of FO is dwindling, failing to meet the growing demand for aquafeeds [8]. About 10–50 kg of fish is required to yield 1 kg of FO [9]. The sustainability issues related to FO production, such as the overfishing of marine resources and environmental degradation, have led to the exploration of alternative lipid sources for aquafeeds [7]. As a result, the aquafeed industry has recently expressed significant interest in exploring and developing alternative sources of terrestrial lipids [10].

Terrestrial oils (TOs) have become viable alternatives to FO because of their higher availability, price stability, and sustainability [11,12,13]. The investigation of TOs from both plant and animal sources has revealed promising alternatives to FO for many aquaculture species [13,14,15,16]. Soybean oil (SBO), rich in linoleic acid, emerged as one of the most widely used TOs in aquafeeds, supporting good growth and health in various fish species [17]. Canola oil, known for its balanced content of omega-3 and omega-6 fatty acids, has demonstrated effectiveness as a replacement in various diets [18]. Palm oil (PLMO), recognized for its high levels of both saturated and unsaturated fats, provides essential energy and fatty acids [19]. Furthermore, chicken fat, as a rendered animal fat, serves as a high-energy alternative appropriate for fish diets [11]. Recently, black soldier fly larvae oil (BSFLO) as a TO source has also been considered as a promising alternative to FO and other plant-based TOs in various fish species [10]. BSFLO is abundant in saturated fatty acids (SFAs) and mono-unsaturated fatty acids (MUFAs), and it also contains a portion of omega-3 fatty acids, which vary based on the nutrients in the culture substrate [20]. Among the SFAs, lauric acid (C12:0) is a prominent component, accounting for up to 52%, and is known for its feeding stimulatory properties [10]. Additionally, palmitic acid (C16:0) and oleic acid (C18:1 n-9) constitute a significant portion of the fatty acids found in larvae, ranging from 12% to 22% and 10% to 25%, respectively [21]. These positive characteristics open new possibilities of using BSFLO in aquafeed for sustainable aquaculture practices.

The physical properties of aquafeed are affected by the dietary ingredient composition, including the dietary oil sources. The inclusion of TOs can affect pellet quality, including feed texture, density, water stability, durability, and dietary attractiveness. High-quality pellets with good water stability, durability, and attractiveness improve feed intake and fish growth, enhancing stock productivity. By contrast, poor pellet stability and durability reduce feed quality and negatively impact biological performance [22,23]. The financial success of culturing fish depends not only on providing nutritionally balanced diets to fish but also on maintaining good physical properties of feed pellets; unfortunately, these issues are often overlooked [24]. For successful aquaculture ventures, these issues need to be considered while formulating feed for various fish species, including Gangetic catfish (*Mystus cavasius*). Currently, there have been no studies examining the effects of various oil sources in diets on the physical properties of feed for Gangetic catfish. Understanding these effects is crucial for developing new feed and ensuring the successful cultivation of this species.

*M. cavasius* is one of the most important aquaculture species in Bangladesh and other countries of the Indian subcontinent. Due to its excellent flavor, this fish is highly sought after by consumers and commands a premium price in the market [25]. It is resistant to extreme environmental conditions, including low oxygen levels and large temperature swings, and this species is carnivorous, primarily consuming small fish and insect larvae for food [26]. Nutritional research on this species is very limited, and no studies have reported on the evaluation of different TOs as an alternative to dietary FO on feed physical properties, growth, feed utilization, and fatty acid profile. Taking these factors into account, this study aimed to identify viable alternatives to FO that could support sustainable aquaculture while ensuring optimal fish growth, health, and nutritional value.

## 2. Materials and Methods

### 2.1. Test Fish and Experimental Design

Initially, a thousand Gulsha catfish fry (*M. cavasius*), each with an average initial weight of 0.45 g, were purchased from a commercial hatchery named Mohananda Agriculture and Fisheries Limited, Habigonj, and transported in an oxygenated polyethylene bag to the wet laboratory at Sylhet Agricultural University’s Department of Aquaculture. Afterward, fish were dipped into potassium permanganate solution and immediately stocked into five glass aquariums (capacity: 100 L) to acclimatize them to tank water environment. During the acclimation period (15 days), the fish were fed a commercial diet containing 40% crude protein twice daily (ACI catfish feed Ltd., Dhaka, Bangladesh). Water quality parameters, including temperature (27 ± 1 °C), dissolved oxygen (>5.5 mg/L), and pH (7.4–7.8), were monitored daily. After 2 weeks, fish were starved for 24 h and individually weighed, and healthy uniform-sized (approx. 0.52 ± 0.02 g) fish were randomly distributed, 45 fish per tank, into 15 glass aquariums (100 L) with continuous aeration. An experimental trial was conducted for 70 days with 5 treatments in triplicate groups.

### 2.2. Formulation of Experimental Diet and Feeding Trial

The basal diet ingredients such as fishmeal (produced from Kachki fish, *Corica soborna*), soybean meal, mustard oil cake, poultry meal, maize meal, vitamin–mineral premix, wheat flour, and vegetable oils like PLMO and SBO were procured from Bandar Bazar local market, Sylhet, and the marine FO and BSFLO were purchased from international market. All ingredients were dried and grinded with a kitchen-type blender into fine powder with a laboratory-scale automated pellet machine in aquaculture feed innovation laboratory of Sylhet Agricultural University. After that, ingredients were screened and measured with a digital balance according to the proportions given in Table 1 for experimental diets.

Vitamins—Vitamin A 14,000,000 I.U., Vitamin D3 300,000 I.U., Vitamin E 3500 mg, Vitamin K3 140 mg, Vitamin C 5000 mg, Vitamin B1 1000 mg, Vitamin B2 700 mg, Vitamin B6 500 mg, Vitamin B12 1800 mcg, Nicotinic Acid 3500 mg, Ca-Pantothanate 1400 mg, Folic acid 100 mg, and Inositol 2500 mg; minerals—Iron (Fe) 700 mg, Zinc (Zn) 2000 mg, Iodine (I) 30 mg, Copper (Cu) 70 mg, Cobalt (Co) 12 mg, Manganese (Mn) 1400 mg, Selenium (Se) 4.8 mg, Calcium (Ca) 250,000 mg, Phosphorus (P) 1000 mg, sodium (Na) 2800 mg, Magnesium (Mg) 5000 mg, and potassium (K) 2500 mg; prebiotic—Fructo-oligosacharides 10,000 mg; Antioxidant—Helmox (BHT/BHA) 10,000 mg and Yeast 60,000 mg; Amino Acids—Lysine 15,000 mg, Tryptophan 200 mg, Threonine 1606 mg, Methionine 20,000 mg, Glycine 2500 mg, Isoleucine 900 mg, and Arginine 0.75 mg.

Five iso-proteinous (40%) and iso-lipidic (11%) experimental diets were prepared using aforementioned ingredients, where the lipid sources were different. For example, Diet 1 (control) contained FO, Diet 2 had SBO, Diet 3 insect oil (BSFLO), Diet 4 PLMO, and Diet 5 mixed oils (50% BSFLO + 25% SBO + 25% PLMO as D5). The diets were prepared by thoroughly mixing all the dry ingredients in a food mixer for 10 min. Oil was added to the dry ingredients and mixed for another 10 min. Water was added gradually (35–40% of the dry ingredients) to the premixed ingredients and mixed for another 10 min. The mixture was then passed through a meat grinder with an appropriate diameter (1.2–2.2 mm) to prepare pellets. The prepared pellets were dried in an oven for 5–6 h at 50 °C and stored in airtight containers at or below 4 °C in the refrigerator until feeding. Furthermore, the proximate composition of the five experimental diets and fatty acid composition of the terrestrial oil and feed samples were analyzed according to AOAC [27] and are shown in Table 2, Table 3, and Table 4, respectively. The diets were administered to the fish at satiation levels twice daily for 70 days at 9 AM and 5 PM.

### 2.3. Measurement of Physiological Parameters of Experimental Diets

The physical parameters such as bulk density, pellet durability index (PDI), and water stability of experimental diets were calculated according to the standard method, with some modification [28,29]. The bulk density was measured by taking a measuring cylinder of 1000 mL, and about 1 kg of test diet was poured. Then, measuring cylinder filled with the feed was subsequently measured using an electronic balance and replicated three times, and the mean value was recorded into an experimental notebook. The bulk density of feed was estimated using following formula:

Bulk density (kg m^−3^) = Measured weight of the feed particles in cylinder (kg)/Total volume occupied (m^3^)

To determine PDI value, 100 g of feed particles of each diet in triplicate groups was collected in a tumbling box tester (Seedburo, Chicago 2 (II), Chicago, IL, USA). Then, the pellets were tumbled continuously for 10 min at a rate of 50 revolutions per minute. After that, a well-designed 1 mm standard-size sieve was used to separate the produced dust from test pellets. Following the period, the weight of the remaining feed sample was measured again with the electronic balance. The PDI of feed was estimated using the following equation:

Pellet durability index, PDI (%) = 100 × (Weight of the remaining feed pellets on the sieve/Initial total weight of pellets before tumbling).

Attractability and palatability of diets were measured according to the methods outlined in previous studies [23,30]. The water stability of each experimental diet was evaluated by calculating the weight of the retrieved whole pellets after 20 min immersion divided by the initial total sample dry weight and then multiplied by 100. After immersion, the fish feed pellets remaining on the wire mesh were dried in a hot air oven at 105 °C for 24 h. The dry weight of the remaining pellets was regarded as the final weight of the sample. The water stability of the fish feed pellets was calculated using the following equation:

Water stability (%) = 100 × (Weight of the retained whole feed pellets after immersion)/(Initial total weight of feed pellets)

### 2.4. Determination of Growth Parameters

At the end of the 70-day feeding trial, fish were fasted for 24 h before final sampling. Fish were anesthetized with ethyl 3-aminobenzoate methanesulfonate (MS-222, Sigma Aldrich, St. Louis, MO, USA) at a concentration of 100 mg/L. The total length and body weight of each fish were measured using a measuring scale and an electronic balance, and the final number of fish in each tank was also recorded. For each replication, growth performance (% weight gain, specific growth rate, condition factor) and feed utilization parameters (feed conversion ratio, feed conversion efficiency, and protein efficiency ratio) were calculated based on standard indices [11].

### 2.5. Proximate Composition Analysis of the Test Diets and Whole Body

Proximate composition of fish whole body and experimental diets were performed in aquatic animal nutrition laboratory in Sylhet Agricultural University following the standard protocol of AOAC (2000) [27].

Moisture was determined by drying at 105 °C for 24 h in a hot air oven, crude protein according to the Kjeldahl method, crude lipid by extraction using N-hexane by VELP Scientifica solvent extractor (made in Italy) and further distillation, and ash by calcination in muffle furnace at 550 °C for 6 h.

### 2.6. Fatty Acid Profiling

Fatty acid profiles of oil sources, experimental diets, and fish whole body were determined using gas chromatography (Model: GC 2010-Plus, Brand: Shimadzu, Origin: Kyoto, Japan) in the laboratory of Bangladesh Council of Scientific and Industrial Research (BCSIR). Hydrolytic method was used for the extraction of fat and fatty acids. Fat was extracted into ether and then methylated to fatty acid methyl esters (FAMEs). Gas chromatography (GC) was used to measure FAMEs quantitatively.

### 2.7. Statistical Analysis

Data are presented as means of three replicates and standard error. All data were subjected to one-way analysis of variance (ANOVA) to test the significant difference between the control and treatment groups. Normality and homogeneity of data were evaluated using Shapiro–Wilk and Levene’s test. Tukey’s Honest Significant Difference (HSD) test was performed to evaluate the variance, considering *p* < 0.05 as significance level. IBM SPSS Statistics version 22.0 was used to perform all statistical tests.

## 3. Results

### 3.1. Measurement of the Physical Properties of the Experimental Diets

The physical characteristics of the pelleted feed containing different oils as lipid sources and fed to Gangetic catfish for 70 days are summarized in Table 5. The physical properties in terms of water stability (%), PDI (%), palatability, and bulk density (kg m^−3^) were significantly different (*p* < 0.05) among the test diets in this study. The same was not true for attractability (%) (*p* > 0.05). Water stability (%) was significantly higher in the mixed oil-based diet (D5) and SBO-based diet (D2), followed by the D3 and D4 diets. In the case of the PDI (%) value, it was significantly higher in the mixed oil-based diet (D5), followed by the SBO-based diet (D2), compared to the other experimental feeds; however, there was no significant difference in PDI (%) among the D1, D3, and D4 diets. However, no statistical difference was observed for the attractability of the feed to the fish among the test diets (*p* > 0.05). The highest (*p* < 0.05) palatability was shown by the D5 and D2 diets, followed by D2, D3, and D1. Finally, the bulk density was significantly higher in the control diet (D1) and PLMO diet (D4). The mixed oil-based diet (D5) had the lowest bulk density, whereas the SBO-based diet (D2) and BSFLO-based diet (D3) had intermediate values.

### 3.2. Growth Performance and Feed Utilization Parameters of the Gangetic Catfish

The growth performance of the Gangetic catfish fed with different test diets is presented in Table 6. There were no significant differences in the IBW of the fish at the start of the trial (*p* > 0.05). The growth and feed utilization parameters of the fish varied significantly (*p* < 0.05) when the fish were fed with different experimental diets. However, the highest weight gain and final body weight were observed in the mixed oil-based diet (D5), while the other test diets had no significant differences. The survival rate of the test fish fed with different diets had no significant differences (*p* > 0.05); however, a numerically lower number of final survivals was observed in the BSFLO-included D3 and D5 diets, and the statistically highest and lowest SGR was observed in the fish fed with the D5 and D2 diets, respectively, whereas the fish in the other diet groups had intermediate values. Moreover, the feed utilization parameters of the test fish were found to be similar in all diet groups. Therefore, feed intake, FCR, FCE, and PER were not significantly influenced (*p* > 0.05) by different treatments.

### 3.3. Condition Factor of Gangetic Catfish Fed Experimental Diets for 70 Days

**Figure 1:**
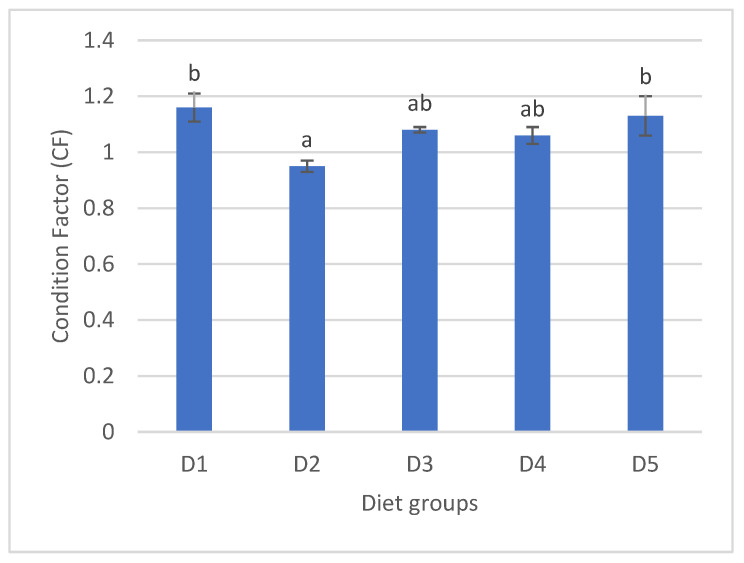
Condition factor of Gangetic catfish fed experimental diets for 70 days. Data were expressed as mean ± S.E.M. from triplicate groups. Values with different letters are significantly different (*p* < 0.05). Condition factor (CF) = 100 × fish weight/(fish length)^3^.

The condition factor (CF) of the experimental fish is presented in Figure 1. There was a significant difference found among the fish in the different experimental groups. The significantly highest (*p* < 0.05) CF was found for the fish fed with the D1 and D5 diets, whereas the D2-fed fish had a significantly lower CF value. The fish fed with the D3 and D4 diets showed intermediate CF values.

### 3.4. Whole-Body Proximate Compositions of Gangetic Catfish

The fish’s overall proximate compositions are shown in Table 7. The crude lipid content in the entire body of the fish fed the various experimental diets varied significantly (*p* < 0.05). The fish fed the D2 and D3 diets had considerably higher and lower crude lipid levels, respectively. The values of the other diets, however, were in the middle. Additionally, the fish’s crude protein, moisture, and ash contents did not differ significantly (*p* > 0.05) between the groups.

### 3.5. Whole-Body Fatty Acid Composition of Gangetic Catfish Fish Fed Test Diets for 70 Days

Table 8 shows the whole-body fatty acid composition of the Gulsha fish fed with different diets. Dietary oil source influences muscle fatty acid composition. The whole-body total saturated fatty acid (∑SFA) contents were significantly low in the SBO-based diets. By contrast, a significantly higher level of ∑SFA was found in the BSFLO-included D5 and D3 diets, while the other D1 and D4 diets had intermediate values. Lauric acid (C12:0) was the highest in the fish fed the BSFLO-included diet (D3), followed by the D5 diet, and the other diets had lower values. Among the mono-unsaturated fatty acids, palmitoleic acid (C16:1) and erucic acid (C22:1) content was significantly higher in the control diet group compared to the TO-based diet groups (*p* < 0.05). By contrast, oleic acid (C18:1) content was significantly lower in the control group (D1) and BSFLO-included group (D3) and significantly higher in the D4 and D5 groups. The ∑MUFA content was significantly higher in the PLMO-containing diet group (D4), followed by the control group, mixed oil group, BSFLO group, and, lastly, the SBO group (*p* < 0.05). The whole-body ᵞ-linolenic acid (C18:3n-6) content was significantly higher in the SBO-based diet (D2), followed by the mixed oil-based diet (D5) and BSFLO-included group (D3), compared to the control group (*p* < 0.05). Higher whole-body α-linolenic acid content was also observed in the SBO-based diet (D2), followed by the BSFLO-based diet (D3) (*p* < 0.05). A significantly higher amount of whole-body total n-3 LC-PUFA content was observed in the fish fed the SBO-based diet (D2), followed by the fish fed the BSFLO-based diet (D3) and control groups (D1); the PLMO-based diet showed a significantly lower content of total n-3 LC-PUFAs. The fish in the FO-based control diet group showed significantly higher whole-body, EPA, DHA, and EPA + DHA content compared to those in the TO-based diet groups (*p* < 0.05).

**Figure 2:**
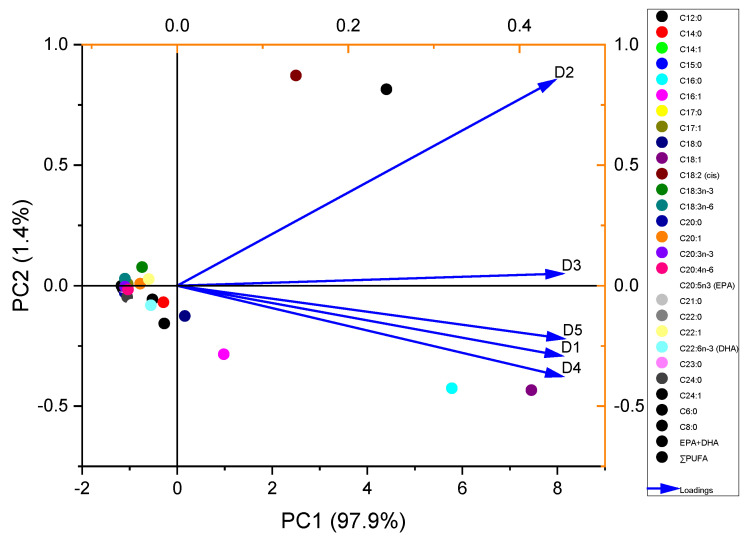
Principal component analysis of whole-body fatty acid composition of Gangetic catfish fed experimental diets for 70 days.

The PCA biplot of the Gangetic catfish’s whole-body fatty acids with varying dietary lipid sources revealed distinct clustering patterns (Figure 2), with PC1 explaining 97.9% and PC2 1.4% of the total variance, collectively accounting for 99.3% of the variability. The fish fed the D1, D4, and D5 diets clustered in the lower-right quadrant, while the fish fed the D2 and D3 diets grouped in the upper-right quadrant, which indicates diet-driven variation in fatty acid profiles. Most fatty acids clustered near the origin, which suggests similar responses across diets, whereas a few showed separation along PC1, reflecting distinct dietary effects. D2 was strongly associated with PC1, while D3 contributed mainly to PC2. Diets D1, D4, and D5 showed comparable fatty acid profiles, characterized by a higher amount of LC-n-3 PUFAs (FO) and palmitic/oleic acids (PLMO), which distinguished them from the SBO and BSFLO diets.

## 4. Discussion

The effects of substituting BSFLO and other vegetable oils for FO as alternative lipid sources on Gangetic catfish (*M. cavasius*) growth, feed utilization parameters, whole-body proximate composition, biometric index, and fatty acid composition, as well as the physiological characteristics of experimental diets, are evaluated for the first time in this study. The formulated test diets were analyzed for proximate composition, fatty acid profile, and gross energy content before initiating the feeding trial to confirm that the target nutritional values were met. The used ingredient proximate composition aligns closely with values reported in previous studies [31,32,33,34,35]. The protein and lipid percentages were approximately 40% and 11%, respectively, which met the nutritional requirements of catfish farming.

The improved physical qualities of aqua feed pellets significantly reduce dust formation and feed waste. However, the characteristics of fish pellets are impacted by formula variations [28]. There are several nutritional studies on the use of various vegetable oils in freshwater and marine species, but there are very few on the use of BSFLO. However, none of these studies have provided information about the feed’s physical characteristics. The effects of different TOs on physical properties may vary from study to study because of differences in the source of nutrients. A large amount of n-3 PUFAs reduces the water and chemical stability of feed pellets [36]. In this study, the water stability of the D1 diet was significantly lower (*p* < 0.05) due to its higher content of n-3 PUFAs compared to the other TO-based diets. On the other hand, the D2 and D5 diets showed higher water stability compared to the FO-based diet because of their reduced n-3 PUFA content. Again, we found that PDI values (%) were lower with increasing n-3 PUFA content in the diet that was categorized as the D1 diet in our study [37]. So, reduced n-3 PUFA content increased the PDI (%) values in the D5 and D3 diets. Our results also showed that palatability was the highest in the mixed oil-based diet (D5) and BSFLO-based diet (D3) groups. It is believed that palatability is the culmination of various dietary factors and that there is a significant relationship between flavor and nutritional value. Feeding with an insect-based diet (frass) also showed increased palatability in channel catfish [38]. In this study, the attractability of the feed ingredients was the highest in the insect oil-based diets (D3 and D5). Thus, Kierończyk regarded this feature as an added benefit of using insect meal, attributing it to the presence of aromatic compounds [39]. Another study also found higher acceptability and palatability for BSFLO-based diets [31]. Their aforementioned diets also showed no noticeable deviation from the FO diet, which indicates that BSFLO had no detrimental effects on palatability [31]. Again, adding insect oil to young Jian carp did not adversely affect palatability [33,35]. On the other hand, other research has found that fish on diets high in medium-chain fatty acids (MCFAs) often exhibit reduced feed intake [40,41]; that is contradictory to our study. But another study found no impact on feed intake for MCFAs [42]. Feed prepared with insect oil incorporation had a lower bulk density, which aligns with our results [43].

After feeding with different TO- and FO-based diets, fish weight increased over fivefold in 70 days. The recorded growth performance in the current study has shown that it is possible to totally replace FO with TOs (SBO, PLMO, BSFLO) in diets for Gangetic catfish without notably impacting growth or feed efficiency. These results align with earlier research findings [44,45,46,47]. Among the groups fed with TO-based diets, the mixed oil-based diet (D5) demonstrated significantly better growth performance than the diets using individual TOs or FO. Earlier research conducted on various fish species, such as amberjack (*Seriola dumerili*) [48] and Juvenile Tench (*Tinca tinca*) [49], found that the dietary inclusion of vegetable blends was completely able to replace FO without any negative effects on growth performance. The better growth performance in the mixed oil group compared to the single terrestrial oil group might be due to a synergistic interaction among BSFLO, SBO, and PLMO, which together provide a more balanced fatty acid profile, improved energy utilization, and enhanced lipid digestibility compared to individual oil sources. This combination may better meet physiological lipid requirements and promote protein sparing, ultimately resulting in optimal growth. Other reasons might be due to the presence of insect oil. Basically, the Gangetic catfish is insectivorous in its natural environment. So, the presence of insect lipids is probably better digested and utilized by this fish, which, in turn, helps to improve growth. In this study, among the TOs, the significantly lowest growth performance was found in the fish fed the SBO diets. Like our study, similar depressed growth was reported in tilapia fish when the dietary total FO was replaced with SBO [49,50] in tilapia diets. By contrast, some other studies reported no adverse effect of using SBO as an alternative to FO and other TOs, like in tilapia [19,51,52], rainbow trout [53], and the large yellow croaker, *Larmichthys crocea* [54]. The variation in these findings might be due to the differences in the basal diet formulation, fish size, species, and dietary lipid level. While soybean oil (SBO) is easily accessible and cost-effective, it has a high content of 18:2n-6, which restricts its use due to the significant decrease in the n-3/n-6 ratio in the final product. Interestingly, in the current study, like the SBO-fed group, the FO-fed groups also showed significantly lower growth performance compared to the groups fed with the other diets. This lower growth performance might be due to the type of FO utilized in the current study. In this study, marine fish oil (FO) was utilized, which has a higher concentration of n-3 LC PUFAs compared to freshwater fish oil. This high level of PUFAs might have negatively affected its utilization performance. Although freshwater fish do not need the highly unsaturated fatty acids in their pre-formed state in their diet in the same way that marine fish do, the fatty acid pattern difference between freshwater FO and marine FO might be one of the reasons for this discrepancy.

In this study, the oil types used did not significantly affect feed utilization performance, which suggests that the use of TOs can effectively replace FO in the diet of Gangetic catfish. A similar non-significant influence of the dietary inclusion of TOs in feed utilization parameters was reported in rainbow trout [53]; large yellow croaker, *Larmichthys crocea* [54]; and mandarin fish, *Siniperca scherzeri* [13]. In this study, the type of oil used did not significantly affect feed intake, which indicates that the diets were similarly palatable and acceptable to the fish. Moreover, a numerically higher feed intake was observed in the BSFLO-included groups (D3, D5) compared to the groups fed with the other diets. This higher feed intake might be associated with the relatively higher amount of lauric acid content of the BSFLO as well as the BSFLO-based experimental diets. The well-established functional features of lauric acid, which include feeding stimulatory effects, have been linked to a number of health advantages. Another study demonstrated that gilthead sea bream (Sparus aurata) exhibited increased feed intake, improved nutrient absorption, enhanced intestinal development, and elevated growth rates when given sodium salt of coconut fatty acid distillate, which is notably high in lauric acid (C12:0) [42]. A recent study also reported increased feed intake in rainbow trout fed terrestrial oil blends that contained BSFLO along with other vegetable oils. The presence of BSFLO finally increases the lauric acid content of the respective diet and helps in improving feed intake [46]. In contrast to the findings of the current study, Luo et al. [55] indicated that the inclusion of 15% coconut oil, which is high in lauric acid, in the diet of rainbow trout led to reduced feed intake and growth when compared to groups that received 15% FO. The differences in the experimental species (salmonids vs. Gangetic catfish), the source of lauric acid supplementation (coconut oil/BSFLO), or the length of the trial (30 days vs. 70 days) could be the cause of this discrepancy.

The type of dietary oil source used in this study had a substantial impact on the CF. Significantly higher CFs were observed in the D1 and D5 groups compared to the D2 diet group, which indicates the relative robustness of the fish in response to the respective diets. A previous study found a reduced CF for an SBO-based diet in rainbow trout compared to FO and other TOs, which completely correlates with the findings of this study [56]. In a separate investigation, the use of another vegetable oil, palm oil (PLMO), was found to decrease the CF in Atlantic salmon (*Salmo salar* L.), which aligns with the results observed in the current study [57]. The reduced condition factor observed in the fish fed a soybean oil-based diet is likely attributable to an imbalance in essential fatty acids, particularly the absence of long-chain n-3 poly-unsaturated fatty acids such as EPA and DHA and a high n-6:n-3 ratio, which can impair growth, lipid metabolism, and overall nutrient utilization while promoting inflammatory responses and oxidative stress. Soybean oil-derived fatty acids are also more readily oxidized for energy rather than retained as body lipids, which results in lower weight gain relative to length. By contrast, the mixed oil diet (D5) likely offers synergistic benefits by combining energy-dense fatty acids with essential and bioactive lipids, optimizing nutrient utilization, promoting protein sparing, and ultimately improving somatic growth and body condition.

In the current study, the overall proximate composition of the major nutrients in the fish’s body was not significantly influenced by the type of dietary oil used, with the exception of whole-body lipid content. Significantly higher and lower whole-body lipid content was observed in the SBO- and BSFLO-based diet groups, respectively. In a previous study, it was found that insect meal or insect oil inclusion significantly reduced the liver lipids in freshwater Atlantic salmon [58], which probably finally helps to reduce whole-body lipids in fish. This is probably because the components derived from insects contain lauric acid. SFAs make up the majority of the fatty acids in BSFL oil (63% of total FA in the current study), with lauric acid, a medium-chain FA, accounting for more than half of this SFA [59,60]. Lauric acid has rapid oxidation and low deposition capacity, as triglycerides possibly lead to a loss of adiposity [61], as shown in mammals [62] and also in fish [63]. Another explanation could be that the group fed with the insect oil expressed more genes linked to the metabolism and breakdown of fat (lipolysis), which reduced the amount of fat that was retained in the tissues overall. Less digestibility is perhaps the other factor. However, neither digestibility nor metabolic gene expression was assessed in the current study; therefore, more research is necessary to support the theory that feeding with insect oil reduces body fat. The significantly higher lipid content in the SBO-based diet might be due to the lower n-3 LC-PUFA content in this diet, which may enhance fatty acid synthesis, inactivate lipoprotein lipase, reduce fatty acid β-oxidation, and enhance triacylglycerol synthesis, which could result in a general increase in lipid deposition in this group [64,65]. Another reason might be the higher fat deposition in the liver and not in the muscle of the fish fed the SBO-based diet. SBO may induce deposition of linoleic acid in the liver. However, overall, the proximate composition values found in this investigation were within the normal ranges previously reported for Gangetic catfish [66].

The fatty acid makeup of fish is widely known to match that of the diet. In the present study, FO was replaced with TOs that had been modified so that the body fatty acid profiles reflect that of the diet. The highest quantity of n-6 PUFA was seen in the SBO-based feed (D2), which resulted in the highest level of whole-body n-6 PUFA in the fish fed the D2 diet. The fish fed the D5 and D3 diets showed a similar pattern in fatty acid composition, with the D5 and D3 diets having the second and third highest concentrations of this fatty acid, respectively. However, fish whole-body linoleic acid content was within the accepted range (10–12%) that has been reported to reduce LDL cholesterol [67]. Moreover, two oil sources, FO and BSFLO, together with Diets D1, D3, and D5, contain a relatively higher amount of α-linolenic acid (18:3n-3) that helps with the synthesis of its own long-chain omega-3 derivatives, specifically EPA and DHA. As a result, the whole-body content of total EPA and DHA (EPA + DHA) was significantly higher in the groups fed with FO and BSFLO and PLMO compared to those receiving SBO and mixed oil. The test fish fed with TO-based diets exhibited higher levels of total n-6 PUFA in their whole body compared to those that received FO-based diets. Previously, similar outcomes were noted in large yellow croaker fed different TOs as dietary lipids [54,68,69]. Due to the presence of a high amount of lauric acid in BSFLO, the diets that contained BSFLO (D3, D5) had a relatively higher amount of this fatty acid, and, after being fed these diets, fish of these respective groups also showed a similar increasing trend in dietary lauric acid content, with a considerably higher lauric acid concentration. Nonetheless, the comparatively lower lauric acid levels in the whole body of the fish fed the D3 and D5 diet groups compared to the amount in the fish fed the other diets may indicate that the fatty acids were readily oxidized to provide energy. Among the terrestrial oil-included diets, whole-body DHA was higher in the fish fed the BSFLO diet compared to those fed the SBO and PLMO diets. A similar effect was found in another study, where SFAs and MUFAs had a sparing impact on DHA and the high amount of SFAs and MUFAs contained in insect oil had the ability to alter fish muscle n-3 LC-PUFA deposition [53]. Moreover, when compared to other fatty acids, DHA was preferentially stored in the Jian carp muscle because its concentration was greater than the corresponding intake from the diet [35]. Conversely, compared to the other diets that were fed to the fish, the D2 diet contains a higher quantity of linoleic acid (20.02%). An overabundance of linoleic acid causes the body to become inflammatory [67]. This increased linoleic acid could be the result of the fish given the D2 diet having lower growth performance. However, we did not conduct any analyses related to inflammation; therefore, further studies are suggested in this regard to support this hypothesis.

Principal component analysis (PCA) clearly differentiated the five dietary groups based on fatty acid composition. PC1 reflected overall fatty acid abundance, showing high positive loadings across all diets and strong associations with major fatty acids and total saturated fatty acids. By contrast, PC2 provided the strongest dietary separation, driven by high linoleic acid (C18:2 cis) content and negative total saturated fatty acids, representing a gradient between unsaturated and saturated profiles. Along with PC2, D2 was associated with higher unsaturated fatty acids, while the other diets clustered toward saturated profiles. PC3 further separated diets based on medium-chain and saturated fatty acids, with D3 contributing the most strongly. PC4 explained less variance but highlighted differences in long-chain poly-unsaturated fatty acids, particularly EPA and DHA. Overall, dietary separation was mainly driven by fatty acid composition, especially the saturated-to-unsaturated balance, rather than total lipid content.

## 5. Conclusions

This study demonstrates that the complete replacement of FO with selected terrestrial oils (SBO, BSFLO, and PLMO) in the diets of Gangetic catfish is nutritionally viable without adverse effects on growth or feed efficiency, demonstrating the species’ capacity to utilize diverse lipid sources. The consistently superior performance of the mixed oil diet highlights the importance of lipid blending, where complementary fatty acid profiles likely act synergistically to optimize energy utilization and protein sparing. Although FO remains essential for maximizing tissue n-3 LC-PUFA levels, particularly EPA and DHA, BSFLO inclusion promoted the accumulation of functional fatty acids such as lauric acid and reduced whole-body lipid deposition, which suggests beneficial metabolic modulation. Comparable MUFA levels in the PLMO and mixed oil treatments further indicate their effectiveness as sustainable energy sources. Collectively, these findings indicate that the strategic blending of terrestrial oils can optimize growth, modulate fatty acid composition, and reduce dependence on finite marine resources. Therefore, mixed oil formulations, followed by BSFLO and PLMO formulations, represent sustainable and functionally effective alternatives to FO in Gangetic catfish feeds.

## Figures and Tables

**Table 1:** Feed formulation of the experimental diets.

Ingredients	D1 (Control)	D2	D3	D4	D5
Fish meal	24	24	24	24	24
Soybean meal	23	23	23	23	23
Mustard oil cake	13	13	13	13	13
Poultry meal	19	19	19	19	19
Maize meal	4	4	4	4	4
Wheat flour	12	12	12	12	12
Vitamin–mineral mix ^‡^	1	1	1	1	1
Oil *	4	4	4	4	4
Total	100	100	100	100	100

* Oil included as follows: D1 = fish oil, D2 = soybean oil, D3 = black soldier fly larvae oil (BSFLO), D4 = palm oil, and D5 = (50% BSFLO + 25% soybean oil + 25% palm oil). ^‡^ Square Aquamix (Square Pharmaceuticals Ltd. Agrovet division, Dhaka, Bangladesh).

**Table 2:** Proximate composition of experimental diet *.

Variables	Diet Groups				
	D1	D2	D3	D4	D5
Moisture (%)	7.93	7.63	6.85	8.58	9.08
Protein (%)	39.99	40.16	39.98	40.33	40.07
Lipid (%)	11.78	11.27	11.86	11.48	11.82
Ash (%)	7.02	7.98	7.20	7.26	7.70
Energy (Kcal/g) **	21.18	20.91	21.17	21.09	21.08

* Oil included as follows: D1 = fish oil, D2 = soybean oil, D3 = black soldier fly larvae oil (BSFLO), D4 = palm oil, and D5 = (50% BSFLO + 25% soybean oil + 25% palm oil). Carbohydrate was calculated by the difference: 100−(protein + lipid + ash+ moisture). ** Calculated using combustion values for protein, lipid, and carbohydrate of 236, 395, and 172 kJ kg^−1^, respectively [11].

**Table 3:** Analyzed fatty acid composition (% of total fatty acids) of different oil sources used in the experimental diets.

Fatty Acid	Oil Sources			
	FO	PLMO	SBO	BSFLO
C10:0	0.003	0.028	ND	0.8122
C12:0	0.12	0.33	ND	42.23
C14:0	6.874	1.05	0.11	7.42
C16:0	20.195	42.02	10.83	12.03
C18:0	4.228	4.45	3.51	1.75
C20:0	0.957	0.33	0.36	ND
ƩSFA	32.377	48.48	14.92	63.75
C16:1n-7	7.998	0.124	0.09	2.53
C18:1n-9	11.446	39.33	24.10	17.10
C20:1n-9	1.809	ND	0.22	ND
ƩMUFA	21.253	39.52	24.25	19.67
C18:2n-6	1.209	10.49	52.42	11.98
C20:4n-6	1.514	ND	0.09	ND
Ʃn6PUFA	2.723	10.49	52.51	11.98
C18:3n-3	0.746	0.349	6.67	2.29
C20:5n-3	14.71	ND	ND	ND
C22:6n-3	11.396	ND	ND	ND
Ʃn-3PUFA	26.852	0.349	6.67	2.29
ƩPUFA	29.575	10.84	59.18	14.27
Ʃn-3PUFA/Ʃn-6PUFA	9.861	0.033	0.127	0.191

Abbreviations: MUFA—mono-unsaturated fatty acid; n-3 PUFA—n-3 poly-unsaturated fatty acid; n-6 PUFA—n-6 poly-unsaturated fatty acid; SFA—saturated fatty acid; EPA—Eicosapentaenoic acid; DHA—Docosahexaenoic acid. “ND” means some fatty acid contents are minor or not detected.

**Table 4:** Analyzed fatty acid (% of total fatty acids) composition of the test diets *.

Fatty Acids	Diet Groups				
	D1	D2	D3	D4	D5
C12:0	0.466	3.014	12.4	0.393	7.45
C14:0	3.976	2.977	3.975	1.462	3.11
C15:0	0.424	0.301	0.41	0.286	0.61
C16:0	22.233	14.89	15.232	24.101	16.92
C17:0	0.378	0.392	0.964	0.545	1.22
C18:0	5.796	5.676	3.973	5.602	4.41
C20:0	0.532	0.472	0.326	0.536	0.41
C22:0	ND	0.516	0.52	ND	0.57
C23:0	0.449	0.237	0.838	0.463	0.94
C24:0	1.789	0.207	0.471	0.33	0.3
∑SFA	36.551	29.511	40.576	33.956	37.89
Monoenes					
C14:1	0.24	ND	0.7	0.337	0.91
C15:1	0.157	ND	0.353	0.079	0.42
C16:1	6.613	1.355	2.67	1.805	2.58
C17:1	0.704	0.234	0.755	0.491	1.84
C18:1	16.783	31.692	17.827	34.114	18.65
C20:1	1.408	0.796	1.553	1.211	1.06
C22:1	7.259	4.706	9.843	8.559	6.92
C24:1	0.48	0.253	0.789	0.423	0.72
∑MUFA	33.644	39.036	34.49	47.019	33.1
C18:2	10.901	26.396	14.474	13.246	17.18
C18:3n-3	2.199	3.047	3.592	2.144	3.55
C20:4n-6	1.248	0.415	1.066	0.673	1.3
C20:5n-3	10.326	0.552	1.76	0.952	2.09
C22:6n-3	4.533	1.042	3.75	2.01	4.29
∑PUFA	29.207	31.452	24.642	19.025	29.02
EPA + DHA	14.859	1.594	5.51	2.962	6.38
∑n-3PUFA	17.058	4.641	9.102	5.106	9.93
∑n-6PUFA	12.149	26.811	15.54	13.919	18.48
Ʃn-3PUFA/Ʃn-6PUFA	1.4	0.173	0.585	0.366	0.537

Abbreviations: MUFA—mono-unsaturated fatty acid; PUFA—BW; SFA—saturated fatty acid; EPA—Eicosapentaenoic acid; DHA—Docosahexaenoic acid. “ND” means some fatty acid contents are minor or not detected. * Oil included as follows: D1 = fish oil, D2 = soybean oil, D3 = black soldier fly larvae oil (BSFLO), D4 = palm oil, and D5 = (50% BSFLO + 25% soybean oil + 25% palm oil).

**Table 5:** Physical properties of experimental diets fed Gulsha during the feeding trial *.

Variables	Diet Groups **				
	D1	D2	D3	D4	D5
Water stability (%)	85.05 ± 0.40 ^a^	91.87 ± 0.17 ^c^	87.14 ± 0.20 ^b^	86.55 ± 0.36 ^ab^	90.74 ± 0.41 ^c^
Pellet durability index (%)	95.26 ± 0.09 ^a^	97.93 ± 0.11 ^bc^	96.50 ± 0.72 ^ab^	96.17 ± 0.05 ^a^	98.46 ± 0.09 ^c^
Attractability (%)	72.47 ± 2.91	72.95 ± 0.48	75.50 ± 7.27	72.57 ± 0.16	79.28 ± 2.54
Palatability	11.27 ± 0.72 ^a^	13.41 ± 0.06 ^bc^	12.71 ± 0.30 ^ab^	12.26 ± 0.55 ^ab^	15.21 ± 0.05 ^c^
Bulk density (kg m^−3^)	496.61 ± 0.28 ^c^	461.52 ± 1.17 ^b^	465.86 ± 0.67 ^b^	496.92 ± 0.26 ^c^	448.0 ± 1.97 ^a^

* Means in the same row that share the same superscript letters are not statistically different (*p* > 0.05; data presented as mean and SE, Completely Randomized Design, one-factor ANOVA, Tukey’s HSD test). The lack of superscript letters indicates no significant differences. ** Oil included as follows: D1 = fish oil, D2 = soybean oil, D3 = black soldier fly larvae oil (BSFLO), D4 = palm oil, and D5 = (50% BSFLO + 25% soybean oil + 25% palm oil).

**Table 6:** Growth and feed utilization performance of Gangetic catfish fed for 70 days *.

Variables	Diet Groups **				
	D1	D2	D3	D4	D5
IBW ^a^	0.52 ± 0.01	0.52 ± 0.02	0.52 ± 0.01	0.52 ± 0.01	0.52 ± 0.01
FBW ^b^	2.74 ± 0.06 ^a^	2.70 ± 0.06 ^a^	2.81 ± 0.04 ^ab^	2.78 ± 0.11 ^ab^	3.08 ± 0.09 ^b^
% WG ^c^	427.12 ± 14.59 ^a^	421.95 ± 8.18 ^a^	439.80 ± 7.49 ^ab^	433.47 ± 9.94 ^ab^	496.60 ± 22.30 ^b^
SGR ^d^	2.08 ± 0.04 ^ab^	2.07 ± 0.02 ^a^	2.11 ± 0.02 ^ab^	2.10 ± 0. 03 ^ab^	2.23 ± 0.05 ^b^
%Survival	82.83 ± 4.36	82.83 ± 5.95	74.27 ± 1.65	88.60 ± 5.70	82.83 ± 8.74
FI ^e^	3.45 ± 0.11	3.50 ± 0.29	3.65 ± 0.01	3.45 ± 0.00	4.01 ± 0.30
FCR ^f^	1.55 ± 0.04	1.60 ± 0.14	1.60 ± 0.03	1.54 ± 0.07	1.56 ± 0.08
FCE ^g^	0.64 ± 0.02	0.63 ± 0.05	0.63 ± 0.01	0.66 ± 0.03	0.64 ± 0.03
PER ^h^	1.61 ± 0.04	1.58 ± 0.13	1.57 ± 0.03	1.63 ± 0.07	1.61 ± 0.09

^a^ IBW—initial body weight (g); ^b^ FBW—final body weight (g); ^c^ WG—percent weight gain (%); ^d^ SGR—specific growth rate (%day−1); ^e^ FI—feed intake (g fish^−1^ 70 days^−1^); ^f^ FCR—feed conversion ratio; ^g^ FCE—feed conversion efficiency; ^h^ PER—protein efficiency ratio. * Means in the same row that share the same superscript letters are not statistically different (*p* > 0.05; Completely Randomized Design, one-factor ANOVA, Tukey’s HSD test). The lack of superscript letters indicates no significant differences. ** Oil included as follows: D1 = fish oil, D2 = soybean oil, D3 = black soldier fly larvae oil (BSFLO), D4 = palm oil, and D5 = (50% BSFLO + 25% soybean oil + 25% palm oil).

**Table 7:** Whole-body proximate compositions (wet weight basis) of Gangetic catfish fed experimental diets for 70 days *.

Variables	Initial ^§^	Diet Groups **				
		D1	D2	D3	D4	D5
Moisture (%)	80.03	72.61 ± 0.40	70.89 ± 0.73	72.81 ± 0.59	73.00 ± 1.30	71.67 ± 0.42
Protein (%)	15.20	16.43 ± 0.39	16.73 ± 0.23	16.61 ± 0.41	15.78 ± 0.77	16.51 ± 0.46
Lipid (%)	2.96	9.29 ± 0.12 ^ab^	10.62 ± 0.56 ^b^	8.91 ± 0.11 ^a^	9.41 ± 0.54 ^ab^	9.66 ± 0.23 ^ab^
Ash (%)	1.82	1.68 ± 0.30	1.48 ± 0.09	1.89 ± 0.33	2.14 ± 0.05	2.06 ± 0.07

^§^ Initial values are not included for statistical analysis. * Means in the same row that share the same superscript letters are not statistically different (*p* > 0.05; Completely Randomized Design, one-factor ANOVA, Tukey’s HSD test). The lack of superscript letters indicates no significant differences. ** Oil included as follows: D1 = fish oil, D2 = soybean oil, D3 = black soldier fly larvae oil (BSFLO), D4 = palm oil, and D5 = (50% BSFLO + 25% soybean oil + 25% palm oil).

**Table 8:** Whole-body fatty acid composition of Gangetic catfish fed test diets for 70 days *.

Fatty Acid	Diet Groups **				
	D1	D2	D3	D4	D5
C6:0	0.28 ± 0.00	0.25 ± 0.02	0.24 ± 0.04	0.33 ± 0.00	0.30 ± 0.04
C8:0	0.45 ± 0.0	0.44 ± 0.07	0.39 ± 0.06	0.51 ± 0.00	0.50 ± 0.06
C12:0	0.47 ± 0.00 ^a^	0.56 ± 0.09 ^a^	6.18 ± 0.22 ^c^	0.68 ± 0.06 ^a^	3.74 ± 0.29 ^b^
C14:0	3.26 ± 0.13 ^b^	2.27 ± 0.02 ^a^	4.71 ± 0.04 ^c^	2.39 ± 0.04 ^a^	3.22 ± 0.23 ^b^
C15:0	0.38 ± 0.03	0.35 ± 0.02	0.39 ± 0.01	0.30 ± 0.00	0.31 ± 0.02
C16:0	24.71 ± 0.12 ^b^	22.62 ± 0.07 ^a^	22.50 ± 0.12 ^a^	26.22 ± 0.06 ^c^	24.55 ± 0.37 ^b^
C17:0	0.49 ± 0.02 ^b^	0.49 ± 0.00 ^b^	0.48 ± 0.00 ^b^	0.38 ± 0.00 ^a^	0.38 ± 0.01 ^a^
C18:0	5.30 ± 0.01	4.02 ± 0.73	4.79 ± 0.05	4.63 ± 0.06	5.04 ± 0.18
C20:0	1.01 ± 0.75	0.30 ± 0.00	0.32 ± 0.02	0.26 ± 0.00	0.21 ± 0.00
C21:0	0.44 ± 0.00 ^a^	0.69 ± 0.02 ^b^	0.75 ± 0.07 ^b^	0.57 ± 0.06 ^ab^	0.66 ± 0.02 ^b^
C22:0	0.20 ± 0.00	0.30 ± 0.00	0.27 ± 0.06	0.29 ± 0.00	0.26 ± 0.07
C23:0	0.33 ± 0.02	0.29 ± 0.02	0.35 ± 0.02	0.31 ± 0.02	0.26 ± 0.00
C24:0	1.30 ± 0.00 ^c^	0.37 ± 0.01 ^a^	0.46 ± 0.01 ^ab^	0.49 ± 0.05 ^b^	0.39 ± 0. 02 ^ab^
∑SFA	38.66 ± 0.83 ^b^	32.97 ± 0.88 ^a^	41.83 ± 0.11 ^c^	37.35 ± 0.20 ^b^	39.82 ± 0.32 ^bc^
Monoenes					
C14:1	0.23 ± 0.01	0.25 ± 0.01	0.23 ± 0.01	0.21 ± 0.01	0.22 ± 0.01
C16:1	10.04 ± 0.42 ^b^	6.09 ± 0.17 ^a^	6.72 ± 0.12 ^a^	7.24 ± 0.70 ^a^	7.70 ± 0.35 ^a^
C17:1	0.36 ± 0.01	0.31 ± 0.01	1.84 ± 0.87	0.27 ± 0.01	0.26 ± 0.01
C18:1	28.34 ± 0.46 ^a^	29.12 ± 0.80 ^ab^	26.67 ± 0.64 ^a^	33.65 ± 0.01 ^c^	32.01 ± 0.91 ^bc^
C20:1	1.66 ± 0.02 ^c^	1.62 ± 0.01 ^bc^	1.84 ± 0.03 ^d^	1.53 ± 0.01 ^b^	1.05 ± 0.05 ^a^
C22:1	2.39 ± 0.01 ^b^	2.39 ± 0.01 ^b^	2.66 ± 0.04 ^b^	2.34 ± 0.03 ^b^	1.02 ± 0.23 ^a^
C24:1	0.20 ± 0.01	0.21 ± 0.03	0.21 ± 0.01	0.16 ± 0.00	0.18 ± 0.04
∑MUFA	43.23 ± 0.87 ^bc^	39.98 ± 0.98 ^a^	40.17 ± 0.27 ^ab^	45.40 ± 0.70 ^c^	42.44 ± 0.30 ^abc^
PUFA					
C18:2	9.92 ± 0.36 ^a^	20.02 ± 0.02 ^d^	11.90 ± 0.06 ^bc^	10.92 ± 0.33 ^ab^	12.20 ± 0.20 ^c^
C18:3n-6	0.26 ± 0.01 ^a^	0.68 ± 0.02 ^d^	0.48 ± 0.01 ^c^	0.39 ± 0.02 ^b^	0.42 ± 0.01 ^bc^
C18:3n-3	1.59 ± 0.07 ^b^	2.30 ± 0.04 ^d^	1.84 ± 0.01 ^c^	1.43 ± 0.05 ^ab^	1.33 ± 0.02 ^a^
C20:3n-3	0.46 ± 0.01	0.41 ± 0.00	0.56 ± 0.09	0.38 ± 0.00	0.39 ± 0.06
C20:4n-6	0.76 ± 0.01	0.56 ± 0.02	0.78 ± 0.12	0.63 ± 0.06	0.57 ± 0.04
C20:5n-3 (EPA)	2.22 ± 0.08 ^b^	0.56 ± 0.01 ^a^	0.67 ± 0.06 ^a^	0.73 ± 0.07 ^a^	0.56 ± 0.13 ^a^
C22:6n-3 (DHA)	3.12 ± 0.01 ^c^	1.86 ± 0.10 ^a^	2.38 ± 0.08 ^b^	2.39 ± 0.16 ^b^	1.79 ± 0.00 ^a^
∑PUFA	18.35 ± 0.54 ^a^	26.39 ± 0.12 ^b^	18.61 ± 0.40 ^a^	16.88 ± 0.54 ^a^	17.28 ± 0.14 ^a^
EPA + DHA	5.35 ± 0.09 ^c^	2.42 ± 0.11 ^a^	3.05 ± 0.13 ^b^	3.12 ± 0.08 ^b^	2.34 ± 0.13 ^a^

Abbreviations: MUFA—mono-unsaturated fatty acid; PUFA—poly-unsaturated fatty acid; SFA—saturated fatty acid; EPA—Eicosapentaenoic acid; DHA—Docosahexaenoic acid. * Means in the same row that share the same superscript letters are not statistically different (*p* > 0.05; data presented as mean and SE, Completely Randomized Design, one-factor ANOVA, Tukey’s HSD test). The lack of superscript letters indicates no significant differences. ** Oil included as follows: D1 = fish oil, D2 = soybean oil, D3 = black soldier fly larvae oil (BSFLO), D4 = palm oil, and D5 = (50% BSFLO + 25% soybean oil + 25% palm oil).

## Data Availability

The data are available from the corresponding author upon request.

## Abbreviations

The following abbreviations are used in this manuscript:

Abbreviation	Full name
ANOVA	One-way analysis of variance
AOAC	Association of Official Agricultural Chemists
BSFLO	Black soldier fly larvae oil
CF	Condition factor
DHA	Docosahexaenoic acid
e.g.,	For example
EPA	Eicosapentaenoic acid
et al.	et alia (and others)
etc.	Etcetera (and other things)
FBW	Final body weight
FCE	Feed conversion efficiency
FCR	Feed conversion ratio
FI	Feed intake
FO	Fish oil
IBW	Initial body weight
MUFA	Mono-unsaturated fatty acid
n-3 PUFA	n-3 poly-unsaturated fatty acid
n-6 PUFA	n-6 poly-unsaturated fatty acid
NRC	National Research Council
PDI	Pellet durability index
PER	Protein efficiency ratio
PLMO	Palm oil
PUFA	Poly-unsaturated fatty acid
SFA	Saturated fatty acid
SGR	Specific growth rate
SBO	Soybean oil
TO	Terrestrial oil
WG	Percent weight gain
